# IgA Immune Complexes Induce Osteoclast-Mediated Bone Resorption

**DOI:** 10.3389/fimmu.2021.651049

**Published:** 2021-07-01

**Authors:** Annelot C. Breedveld, Melissa M. J. van Gool, Myrthe A. M. van Delft, Conny J. van der Laken, Teun J. de Vries, Ineke D. C. Jansen, Marjolein van Egmond

**Affiliations:** ^1^ Department of Molecular Cell Biology and Immunology, Amsterdam UMC, Vrije Universiteit Amsterdam, Amsterdam, Netherlands; ^2^ Amsterdam institute for Infection and Immunity, Amsterdam UMC, Amsterdam, Netherlands; ^3^ Department of Rheumatology, Amsterdam UMC, Amsterdam, Netherlands; ^4^ Department of Periodontology, Academic Centre for Dentistry Amsterdam (ACTA), Vrije Universiteit Amsterdam and University of Amsterdam, Amsterdam, Netherlands; ^5^ Department of Surgery, Amsterdam UMC, Vrije Universiteit Amsterdam, Amsterdam, Netherlands

**Keywords:** ACPA, autoantibodies, bone resorption, IgA, osteoclast, rheumatoid arthritis

## Abstract

**Objective:**

Autoantibodies are detected in most patients with rheumatoid arthritis (RA) and can be of the IgM, IgG or IgA subclass. Correlations between IgA autoantibodies and more severe disease activity have been previously reported, but the functional role of IgA autoantibodies in the pathogenesis of RA is ill understood. In this study, we explored the effect of IgA immune complexes on osteoclast mediated bone resorption.

**Methods:**

Anti-citrullinated peptide antibody (ACPA) and anti-carbamylated protein (anti-CarP) antibody levels of the IgA and IgG isotype and rheumatoid factor (RF) IgA were determined in synovial fluid (SF) of RA patients. Monocytes, neutrophils, and osteoclasts were stimulated with precipitated immune complexes from SF of RA patients or IgA- and IgG-coated beads. Activation was determined by neutrophil extracellular trap (NET) release, cytokine secretion, and bone resorption.

**Results:**

NET formation by neutrophils was enhanced by SF immune complexes compared to immune complexes from healthy or RA serum. Monocytes stimulated with isolated SF immune complexes released IL-6 and IL-8, which correlated with the levels of ACPA IgA levels in SF. Osteoclasts cultured in the presence of supernatant of IgA-activated monocytes resorbed significantly more bone compared to osteoclasts that were cultured in supernatant of IgG-activated monocytes (p=0.0233). Osteoclasts expressed the Fc receptor for IgA (FcαRI; CD89) and Fc gamma receptors. IgA-activated osteoclasts however produced significantly increased levels of IL-6 (p<0.0001) and IL-8 (p=0.0007) compared to IgG-activated osteoclasts. Both IL-6 (p=0.03) and IL-8 (p=0.0054) significantly enhanced bone resorption by osteoclasts.

**Conclusion:**

IgA autoantibodies induce release of IL-6 and IL-8 by immune cells as well as osteoclasts, which enhances bone resorption by osteoclasts. We anticipate that this will result in more severe disease activity in RA patients. Targeting IgA-FcαRI interactions therefore represents a promising novel therapeutic strategy for RA patients with IgA autoantibodies.

## Introduction

Rheumatoid arthritis (RA) is a heterogeneous chronic inflammatory disease that is mainly characterized by swelling and pain in the joints. Approximately 0.5-1% of the population is affected and the incidence in women is higher compared to men. Distinct clinical phenotypes of RA can develop, which are thought to be a consequence of interactions between genetic and environmental factors (1). Autoantibodies often appear long before any signs of joint inflammation emerge (2–5). The majority of RA patients develop antibodies against the Fc region of IgG (rheumatoid factor; RF). However, RF autoantibodies can also be found in patients with other rheumatic diseases and in non-rheumatic patients. Only IgM RF (and not IgG RF) is used in serological tests for RA. Antibodies against post-translationally modified proteins including anti-citrullinated protein antibodies (ACPAs) and anti-carbamylated protein (anti-CarP) antibodies are highly specific. ACPAs can be locally produced by plasma cells in joints of RA patients and are detected in sera of up to 70% of RA patients (6). They recognize citrullinated proteins or peptides, which can be found throughout the body including cartilage and bone. Anti-CarP antibodies are detected in sera of 45% of RA patients and recognize carbamylated proteins (7).

Currently, presence of ACPA and RF in serum are the most commonly used diagnostic markers for RA. Tested antibodies are predominantly of the IgG and IgM isotype. RF, ACPA and anti-CarP antibodies have been associated with bone loss in patients with established RA, suggesting a pathogenic role (3, 7, 8). Notably, bone loss has also been reported in ACPA-positive individuals who did not have joint inflammation, suggesting an adverse effect of ACPA on bone (9).

Bone remodeling is a process in which osteoblasts generate new bone and osteoclasts resorb bone to maintain bone homeostasis. This balance is lost in RA patients (10). Osteoclasts are large multinucleated cells, which form by fusion of mononuclear progenitors from the CD14^+^ monocyte/macrophage lineage. Differentiation, survival and activity of osteoclasts depends on the presence of receptor activator of NF-κB ligand (RANK-L) and macrophage colony-stimulating factor (M-CSF) (11). Osteoclasts and their precursors express RANK, which interacts with RANK-L. The formation of osteoclasts is enhanced in an inflammatory environment by several cytokines, including interleukin (IL)-1β, IL-6, IL-8 and, in low doses by transforming growth factor (TGF)-β, which triggers RANK expression on osteoclast precursors (12, 13). Increased levels of these cytokines have been found in synovial fluid of RA patients and have been reported to contribute to RA disease activity (14). These cytokines can be produced by inflammatory cells in the joint, such as monocytes and macrophages. Additionally, neutrophils are abundantly present and have been reported to express RANK-L. Their contribution in osteoclast activation is however debated (15, 16). In the presence of autoantibodies neutrophils can form NETs and contribute to tissue damage by releasing their granule content (17).

Interestingly, neutrophils are particularly potently activated by IgA antibodies (18). Although they are not routinely tested in RA, more attention has been payed to IgA autoantibodies in RA in the last decade. A considerable proportion (~30%) of RA patients is negative for ACPA IgG and RF IgM (19). About one third of ACPA IgG and/or RF IgM seronegative patients had IgA autoantibody levels in their sera (RF, ACPA or anti-CarP) (3, 7, 19, 20). Several studies have reported a correlation between the amount of IgA and disease severity in RA patients (21). Elevated levels of serum RF IgA were associated with extra-articular manifestations in RA patients and worse disease outcome (22, 23). Moreover, patients having erosions in the bones of their hands had higher serum levels of RF IgA (24). Similarly, presence of anti-CarP IgA was associated with more severe disease (7). Nonetheless, the role of IgA autoantibodies on bone erosion is currently unknown. In this study we investigated the effect of IgA complexes and immune complexes isolated from synovial fluid of RA patients on bone resorption.

## Materials and Methods

### Patients

Synovial fluid from the knee (n=26) and serum (n=20) of RA patients was anonymously collected during their visit to the rheumatology department at the Leiden University Medical Center or the Amsterdam University Medical Center. Patients gave their written informed consent in accordance with the guidelines of the Medial Ethical Committee of the Leiden University Medical Center (reference no. B15.003) or the Amsterdam University Medical Center (reference no. 2013.234).

### Precipitation of Immune Complexes from Synovial Fluid and Serum

Synovial fluids or sera were centrifuged at 3300x*g* for 5 minutes before use. Supernatants were mixed with an equal volume of 10% PEG6000 (Sigma Aldrich, St. Louis, MO) and incubated overnight at 4°C. Precipitates were collected after centrifugation for 10 minutes at 9500x*g* and resuspended in phosphate buffered saline (PBS; B.Braun, Melsungen, Germany) to the original volume of the synovial fluid/serum.

### Serum Collection and Isolation of Human Monocytes and PMN

Serum was collected from healthy donors. Monocytes and polymorphonuclear leukocytes (PMNs) were isolated from peripheral blood obtained from healthy donors or from buffy coats (Sanquin, Amsterdam, The Netherlands). Monocytes were isolated using MACS CD14 MicroBeads (Miltenyi Biotec, Bergisch Gladbach, Germany) according the manufacturer’s protocol. In brief, peripheral blood mononuclear cells (PBMCs) and PMNs were collected after Lymphoprep (Axis-Shield, Oslo, Norway) density gradient centrifugation. PBMCs were washed in PBS and incubated with MACS CD14 MicroBeads after which CD14^+^ monocytes were isolated. Erythrocytes present in the PMN fraction were lysed with ammonium chloride buffer (155mM NH4Cl, 10mM KHCO3 and 0.11mM EDTA). Cells were resuspended in RPMI 1640 (Gibco BRL, Paisley, UK) supplemented with 1% heat-inactivated fetal calf serum (FCS), glutamine and antibiotics. PMN were incubated for 30 minutes (37°C, 5% CO2) prior to use.

### Coating of Beads With BSA, IgA or IgG

Latex beads (carboxylate-modified polystyrene, green fluorescent (1.0 μm); Sigma Aldrich) were washed with sterile 2-(N-morpholino) ethanesulfonic (MES) buffer (30 mM, pH 6.1) and resuspended in MES buffer with 2 mg/ml endotoxin free bovine serum albumin (BSA) (Akron Biotech, Boca Raton, FL), human serum IgA (Cappel, MP Biomedicals, Santa Ana, CA) or human serum IgG (Sigma Aldrich) in the presence of N-(3-Dimethylaminopropyl)-N’-acid ethylcarbodimide hydrochlorid (Sigma Aldrich) and incubated overnight at room temperature in an overhead shaker. Beads were resuspended in sterile PBS containing 0.5% BSA.

### Monocyte Stimulation With Immune Complexes

CD14^+^ monocytes (2x10^6^/ml) were stimulated for 24 hours with BSA-, IgA-, or IgG coated beads in an effector target (ET) ratio of 1:100. Alternatively, 1x10^5^ monocytes were stimulated for 24 hours with 10, 20 or 40 μl of immune complexes isolated from synovial fluid of RA patients. Supernatants were harvested and stored at -20°C.

### NETs Release After Stimulation With Immune Complexes

PMNs (1x10^5^) were incubated in RPMI with 1% FCS in black 96 well plates (FLUORTRAC 200, Greiner Bio-One) with 10 μl precipitated immune complexes from synovial fluid or serum from RA patients or healthy controls for 3 hours at 37°C. Release of extracellular DNA was detected by adding nucleic acid label SYTOX green (2,5 μg/ml; Invitrogen Life Technologies). Optical density was measured using a fluorimeter (FLUOstar/POLARstar BMG Labtech GmbH, Offenburg, Germany) at 480nm excitation and 520nm emission.

### Dectection of Nuclei, αVβ3 & Fc Receptors on Osteoclasts

Isolated CD14^+^ monocytes (2x10^6^/ml) were resuspended in minimum essential medium, α modification (α-MEM) (Gibco BRL), supplemented with 10% FCS, 25 ng/ml M-CSF (ImmunoTools, Friesoythe, Germany), and 50 ng/ml RANKL (BioLegend, San Diego, CA). Cells were seeded in 3 well ibidi slides (removable chamber) (Ibidi, Gräfelfing, Germany) for microscopy imaging or 48 well plates for flow cytometry analysis and cultured for 7 days at 37°C, 5% CO_2_. Medium was changed twice weekly. Tartrate-resistant acid phosphatase (TRAcP) activity in osteoclasts on ibidi slides was measured with the leukocyte acid phosphatase kit 386A (Sigma Aldrich) according to the manufacturer’s instruction and as previously described (25) with slight modifications. Cells were washed with PBS, fixed using 1% paraformaldehyde (PFA) for 10 minutes, washed with MilliQ and incubated with 1 mM tartrate solution for 60 minutes at 37°C. Cells were stained with anti-human CD14-AF488 (Clone HCD14, Biolegend), anti-human CD51/CD61-APC (αVβ3, Clone 23C6, Miltenyi Biotec), and anti-human CD89-PE-Cy7 (Clone A59, Biolegend) for 1 hour at room temperature. Nuclei and cell membranes were stained with 4′,6-diamidino-2-phenylindole (DAPI) and Wheat germ agglutinin (WGA)-AF488 (both ThermoFisher, Waltham, MA), respectively. Osteoclasts were visualized with microscopy (Leica DM6000, Leica, Wetzlar, Germany).

For flow cytometry analysis osteoclasts were detached from the 48 wells plates detached by scraping after 7 day culture. Cells were washed in PBS and stained with fixable viability dye eFluor506 (ThermoFisher) for 20 minutes on ice after which cells were blocked in 5% PBSA for 30 minutes. Cells were stained with anti-human CD14-AF488 (Clone HCD14, Biolegend) and anti-human CD51/CD61-APC (Clone 23C6, Miltenyi Biotec) for 30 minutes on ice. To investigate Fc receptor expression, cells were stained with anti-human CD14-AF488 combined with anti-human CD89-PE-Cy7 (Clone A59, Biolegend), anti-human CD16-APC-eFluor780 (Clone 3G8, ThermoFisher), anti-human CD32-PE-Cy7 (Clone 6C4, ThermoFisher) or anti-human CD64-APC (Clone 10.1, ThermoFisher) for 30 minutes on ice. To determine the number of nuclei, cells were stained with 5 μg/ml Hoechst 33342 (ThermoFisher) for 20 minutes at room temperature. Integrin and Fc receptor expression was analyzed with flow cytometry (LSRFortessa™ X-20, BD Biosciences). Data was processed in FlowJo version 10.6.2. (Tree Star, Inc., Ashland, OR).

### Bone Resorption Assay

Bovine cortical bone was cut in slices of 0.5 mm thick and cut to fit in 96 well plates. Bone slices were sonicated, washed with PBS and incubated in α-MEM (Gibco BRL) for 30 minutes at 37°C after which 0.5x10^6^ CD14^+^ monocytes resuspended in α-MEM supplemented with 10% FCS, 25 ng/ml M-CSF (ImmunoTools) and 50 ng/ml RANKL (BioLegend) were seeded in 96 well plates for 21 days. Supernatant of unstimulated monocytes or monocytes that had been stimulated with BSA-, IgA-, or IgG-coated beads for 24 hours was added (1:1) when indicated. Supernatants and medium were replaced twice weekly and when indicated, medium was supplemented with 25 ng/ml IL-6 (ImmunoTools), 10 ng/ml IL-8 or 5 ng/ml TNF-α (both Peprotech, Rocky Hill, NJ). After 21 days bone slices were stored in water (4°C) and bone resorption was visualized as previously described (26). In brief, bone slices were sonicated for 30 minutes in 10% NH_4_OH, washed in distilled water, shortly dried on filter paper and transferred to new wells containing water saturated aluminium potassium sulfate dodecahydrate (Merck Millipore, Burlington, MA) for 10 minutes. Bone slices were washed twice in distilled water, after which they were washed once under a strong current of distilled water and dried between filter paper. Bone slices were stained with Coomassie brilliant blue solution (PhastGel^®^ blue R, PlusOne Coomassie tablets, Pharmacia, Uppsala Sweden) for 1 minute, and dried between filter paper. Bone resorption pits were visualized with an inverted microscope (Nikon TE-300, Nikon, Tokyo, Japan) and quantified with Image J version 1.49v.

### Purification and Stimulation of CD14 Negative Osteoclasts

Generated osteoclasts (7 day culture) were scraped from 48 well plates and incubated with MACS CD14 MicroBeads human (Miltenyi Biotec) according the manufacturer’s instruction. The flow through containing CD14 negative cells was collected. To confirm purity of CD14 negative cells, cells were washed in 0.5% PBSA, blocked in 5% PBSA for 30 minutes on ice and stained with anti-human CD14-AF488 (Clone HCD14, Cat#325610, Biolegend) for 30 minutes on ice. Cells were measured with flow cytometry (LSRFortessa™ X-20, BD Biosciences) and data was analyzed with FlowJo version 10.6.2 (Tree Star, Inc.). CD14 negative cells were resuspended in α-MEM supplemented with 10% FCS, 25 ng/ml M-CSF (ImmunoTools) and 50 ng/ml RANKL (BioLegend) and plated in 48 well plates for 24 hours. Medium was refreshed and cells were stimulated with 5 μl of BSA-, IgA-, or IgG-coated beads or 40 μl of immune complexes isolated from synovial fluid of RA patients for 24 hours, after which supernatants were collected and stored at -20°C. To determine the morphology of the sorted osteoclasts, TRAcP activity was measured and nuclei were stained with DAPI as described above.

### IL-6 and IL-8 ELISA

Levels of IL-6 and IL-8 were measured with enzyme-linked immunosorbent assay (ELISA) following the manufacturer’s protocol (Human uncoated ELISA kits, Invitrogen, ThermoFisher).

### Anti-Citrullinated Protein Antibody (ACPA) ELISA

ACPA IgA and IgG levels were determined in synovial fluids of RA patients according to the protocol previously described (20) with minor adaptations. Microtiter ELISA plates (Nunc MaxiSorp™ flat-bottom plates; ThermoFisher) were coated with 50 μl of 1 μg/ml streptavidin (ThermoFisher) and incubated overnight at 4°C after which 50 μl of 1 μg/ml biotinylated CCP2-cittruline or CCP2-arginine (kindly provided by Dr. J.W. Drijfhout, Department of Immunohematology and Blood Transfusion (Leiden University Medical Center)) was added for 1 hour at RT. Synovial fluid (1:10 diluted) was added for 1 hour at 37°C. Wells were washed and incubated with F(ab’)2 goat anti-human IgA-HRP (1:4000, ThermoFisher) or F(ab’)2 goat anti-human IgG-HRP (1:4000, ThermoFisher) for 1 hour at 37°C. Fifty μl of 3,3′,5,5′-Tetramethylbenzidine (TMB) substrate was added after which the reaction was stopped with 50 μl of sulfuric acid (10% H_2_SO_4_). Absorbance was measured with a microplate reader (Bio-Rad, Berkeley, CA) at 450nm.

### Anti-Carbamylated Protein (anti-CarP) Antibody ELISA

Anti-Carp IgA and IgG were determined in synovial fluids of RA patients according to protocols previously described (7) with minor adaptations. Microtiter ELISA plates (ThermoFisher) were coated with 50 μl of 10 μg/ml carbamylated bovine serum albumin (Ca-BSA) or non-modified BSA and incubated overnight at 4°C after which wells were blocked with 1% PBSA for 6 hours at 4°C. Synovial fluid (1:10 diluted) was added for 1 hour overnight at 4°C. Wells were washed and incubated with F(ab’)2 goat anti-human IgA-HRP (1:4000, ThermoFisher) or F(ab’)2 goat anti-human IgG-HRP (1:4000, ThermoFisher) for 1 hour at 37°C. Fifty μl of TMB substrate was added after which the reaction was stopped with 50 μl of 10% H_2_SO_4_. Absorbance was measured with a microplate reader (Bio-Rad, Berkeley, CA) at 450nm.

### Rheumatoid Factor (RF) Antibody ELISA

RF IgA levels were determined in synovial fluid of RA patients. Microtiter ELISA plates (ThermoFisher) were coated with 50 μl of 50 μg/ml human serum IgG (Sigma Aldrich) and incubated overnight at 4°C after which wells were blocked with 1% PBSA for 1h at 37°C. Synovial fluid (1:10 diluted) was added for 1 hour at 37°C. Wells were washed and incubated with F(ab’)2 goat anti-human IgA-HRP (1:4000, ThermoFisher) for 1 hour at 37°C. Fifty μl of TMB substrate was added after which the reaction was stopped with 50 μl of 10% H_2_SO_4_. Absorbance was measured with a microplate reader (Bio-Rad, Berkeley, CA) at 450nm.

### Statistical Analysis

Statistical analysis were performed with GraphPad Prism version 8.2.1 (GraphPad, San Diego, CA). Statistical differences were determined with unpaired Student two-tailed t tests (two groups) or one-way ANOVA (more than two groups) for normally distributed data. Mann-Whitney U tests (two groups) or Kruskall-Wallis tests (more than two groups) were applied to not normally distributed data. Correlations were determined with nonparametric Spearman correlation coefficients. P values <0.05 were considered statistically significant.

## Results

### Autoantibodies in Synovial Fluid Induce Immune Cell Activation

**Figure 1:**
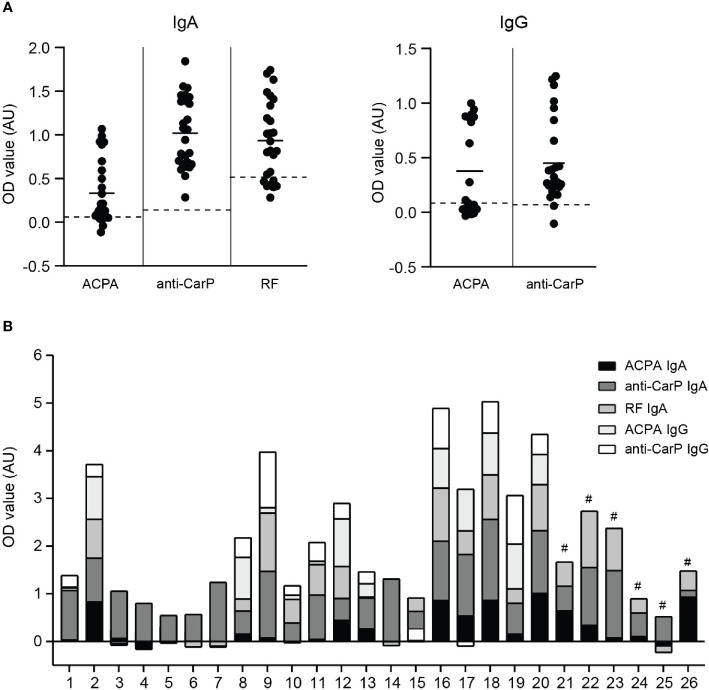
IgA and IgG autoantibody levels in synovial fluid of RA patients **(A)** left panel; ACPA, anti-CarP and RF IgA levels and right panel; ACPA and anti-CarP IgG levels in synovial fluid of RA patients (expressed as OD value). Dotted lines represent blanc OD values of each independent ELISA. **(B)** Synovial fluid profile of ACPA, anti-CarP and RF IgA and IgG levels of 26 RA patients [# IgG levels not determined]. AU, arbitrary units.

To investigate whether local autoantibodies have a potential pathogenic role in RA, we first determined ACPA and anti-CarP IgA and IgG levels in synovial fluid of 26 RA patients (**Figure 1A**). Since we previously demonstrated that RF IgA levels were significantly elevated in RA patients (27), we therefore additionally determined RF IgA in this study. Both IgA and IgG autoantibody levels showed great inter-patient variation. For every patient a profile of IgA and IgG autoantibodies present in their synovial fluid was created (**Figure 1B**).

**Figure 2:**
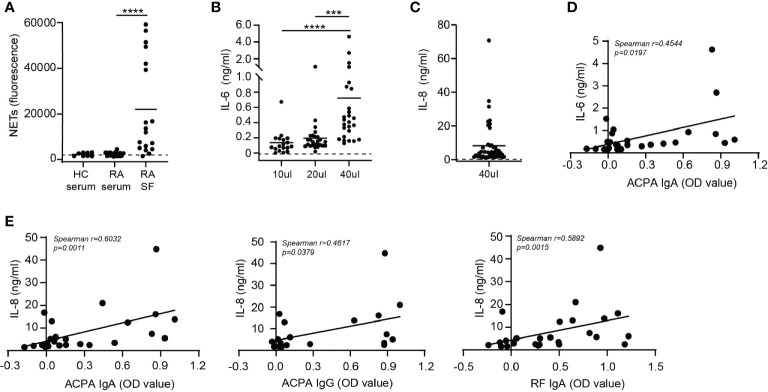
Cytokine release after synovial fluid immune complex stimulation correlates with IgA autoantibody levels in synovial fluid. **(A)** NET release by neutrophils stimulated with immune complexes isolated from healthy control (HC) serum, rheumatoid arthritis (RA) serum or synovial fluid of RA patients (RA SF). Dotted line represents unstimulated neutrophils. **(B, C)** Monocyte **(B)** IL-6 and **(C)** IL-8 release (pooled data from 2 independent experiments) after stimulation with immune complexes isolated from RA SF. Dotted lines represent unstimulated monocytes. **(D)** Correlation between immune complex-induced monocyte IL-6 release with ACPA IgA levels. **(E)** Correlations between monocyte IL-8 release induced by immune complexes isolated from RA SF with (left) ACPA IgA levels, (middle) ACPA IgG levels or (right) RF IgA levels Average cytokine release is displayed (*n*=2). Data was analyzed using two way ANOVA with Bonferroni post-hoc or using the Spearman correlation coefficient; ***p ≤ 0.001, ****p ≤ 0.0001.

When neutrophils were stimulated with immune complexes isolated from synovial fluid of RA patients they formed NETs (**Figure 2A**). The level of NET formation varied greatly between samples. Immune complexes isolated from serum of either healthy controls or RA patients did not induced NET release (**Figure 2A**), which may have been due to lower concentrations of complexes in serum compared to SF. Of note, concentration of immune complexes in sera and SF of RA patients could not be determined due to limited patient material. NET release induced by SF immune complexes significantly positively correlated with ACPA IgA, ACPA IgG and anti-CarP IgG levels present in SF (**Supplementary Figures 1A, B**). Neither anti-CarP IgA nor RF IgA levels in SF correlated with NET release (**Supplementary Figures 1B, C**).

Next, monocytes were activated with immune complexes isolated from synovial fluid of RA patients and IL-6 production was determined (**Figure 2B**). Monocytes were not stimulated with immune complexes isolated from serum of RA patients as neutrophils were not activated by serum immune complexes. Addition of a higher volume of SF immune complexes to monocytes resulted in increased IL-6 release. Furthermore, monocyte IL-8 production after stimulation with 40 μl of immune complexes was enhanced compared to unstimulated monocytes (**Figure 2C**) and positively correlated with IL-6 release (p=0.0003) (**Supplementary Figure 2A**). Immune complexes are large aggregates of autoantibodies and contain multiple antibody isotypes. ACPA IgA levels significantly correlated (p=0.0197) with monocyte IL-6 release induced by these immune complexes (**Figure 2D**). Anti-CarP IgA and RF IgA followed a similar trend, but did not significantly correlate with IL-6 release (**Supplementary Figure 2B**). Neither ACPA nor anti-CarP IgG autoantibodies correlated with IL-6 release (**Supplementary Figure 2C**). Significant positive correlations between ACPA IgA (p=0.0011), ACPA IgG (p=0.0397) and RF IgA (p=0.0015) with monocyte IL-8 release induced by synovial fluid immune complexes were found (**Figure 2E**). While anti-CarP IgA and anti-CarP IgG did not significantly correlate with monocyte IL-8 release (**Supplementary Figure 2D**).

### Osteoclasts formation and activity

**Figure 3:**
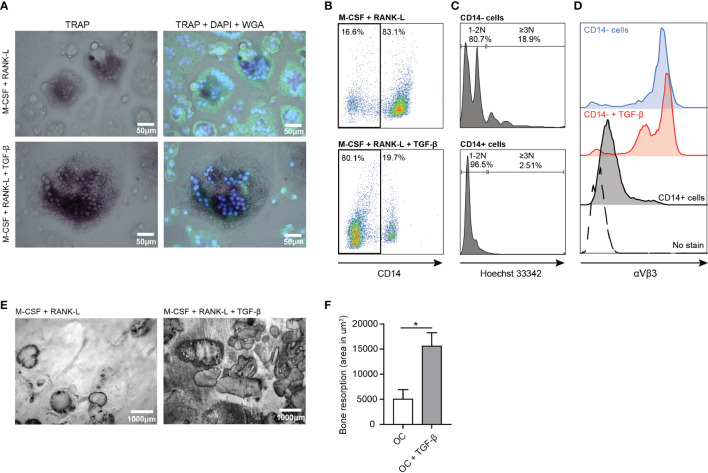
Enhanced osteoclast formation and activity in presence of TGF-β. **(A)** Osteoclast TRAcP expression (purple) in presence of M-CSF and RANK-L (upper left panel) and in presence of M-CSF, RANK-L and TGF-β (lower left panel) after 7 days culture. [Blue (DAPI) = nuclei, green (WGA) = cell membrane]. **(B)** CD14 expression on osteoclasts in absence (upper panel) or presence of TGF-β (lower panel) after 7 days culture. **(C)** Number of nuclei present in CD14- (upper panel) and CD14+ (lower panel) cells after 7 days of culture in presence of M-CSF and RANK-L. **(D)** Expression of αVβ3 on formed osteoclasts in absence (blue (top) histogram) or presence of TGF-β (red (second) histogram) after 7 days culture. Residing CD14^+^ cells (black histogram) and non-stained cells (dotted histogram) served as controls. **(E)** Bone resorption by human osteoclasts after 21 days in presence of M-CSF and RANK-L (left panel) or M-CSF, RANK-L and TGF-β (right panel) (representative of *n*=2 experiments). **(F)** Quantification of bone resorption by osteoclasts in absence of TGF-β (white bar) or presence of TGF-β (grey bar) presented as area in μm^2^ bone resorption. Data was analyzed using unpaired student’s two-tailed T test; *p ≤ 0.05.

*In vitro* generated osteoclasts (OCs) are defined as large multinucleated cells, which express the enzyme tartrate-resistant acid phosphatase (TRAcP) and can resorb bone (28). When osteoclasts mature, CD14 expression is lost and the expression of TRAcP increases (29, 30). We generated multinucleated cells by culturing CD14^+^ monocytes for 7 days in the presence of M-CSF and RANK-L which expressed TRAcP (**Figure 3A**). We defined osteoclasts as multinucleated CD14 negative cells with at least 3 nuclei and expressing high levels of TRAcP and αVβ3. The αVβ3 integrin enables the osteoclast to connect to the bone matrix (31). Not all multinucleated cells differentiated into osteoclasts as TRAcP and the αVβ3 integrin were absent. These cells were likely multinucleated giant cells of macrophage origin. Since TGF-β is an important modulator of osteoclasts, we analyzed whether TGF-β influenced osteoclast formation. Addition of TGF-β to the 7 day culture resulted in formation of larger OCs with increased TRAcP expression (**Figure 3A**). Moreover, presence of TGF-β resulted in the formation of more CD14^-^ cells (~80%) compared to absence of TGF-β (~20%) (**Figure 3B**). Approximately 19% of the CD14^-^ population was classified as mature osteoclasts as they contained 3 or more nuclei (**Figure 3C**). The formed OCs expressed high levels of the αVβ3 integrin independently of TGF-β presence (**Figure 3D**).

OCs cultured on bone slices for 21 days in presence of M-CSF and RANK-L resorbed bone as visualized by formed bone resorption pits and tracks (**Figure 3E**, left panel). Presence of TGF-β significantly increased bone resorption by OCs (**Figure 3E**, right panel). OCs in absence of TGF-β resorbed ~5000 μm^2^ bone, while OCs in presence of TGF-β resorbed significantly more bone (~15000 μm^2^) (p=0.0445) (**Figure 3F**).

### IgA Activated Monocytes Induce Osteoclast-Mediated Bone Resorption

**Figure 4:**
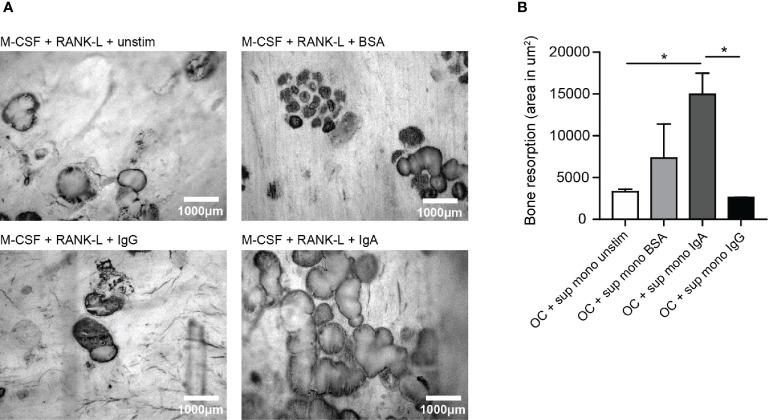
Significantly enhanced bone resorption in presence of supernatant of IgA-activated monocytes. **(A)** Bone resorption by osteoclasts in the presence of supernatant of unstimulated monocytes (upper left panel), BSA-stimulated monocytes (upper right panel), IgG-activated monocytes (lower left panel), or IgA-activated monocytes (lower right panel). **(B)** Quantification of bone resorption (area in um^2^) induced by osteoclasts in presence of supernatant of unstimulated monocytes (white bar), BSA-activated monocytes (light grey bar), IgA-activated monocytes (dark grey bar) or IgG-activated monocytes (black bar). Data was analyzed using one way ANOVA with Tukey post-hoc; *p ≤ 0.05.

Supernatant of (activated) monocytes was added to osteoclasts which were plated on bone slices to study its effect on bone resorption. Unstimulated and BSA-activated monocytes supernatant served as control conditions and only induced little bone resorption by osteoclasts (respectively ~3300 μm^2^ and ~7300 μm^2^) as visualized by dark bone resorption pits (**Figure 4A**). Supernatant of monocytes that had been activated with IgA complexes enhanced osteoclast induced bone resorption compared to supernatant of IgG-activated monocytes (**Figure 4A**). OCs in presence of supernatant of IgA-activated monocytes resorbed on average ~15000 μm^2^ of the bone slices, while supernatant of IgG-activated monocytes only induced resorption of ~2600 μm^2^ bone by OCs (p=0.0233) (**Figure 4B**).

### Osteoclasts Express FcαRI and Are Activated by IgA

**Figure 5:**
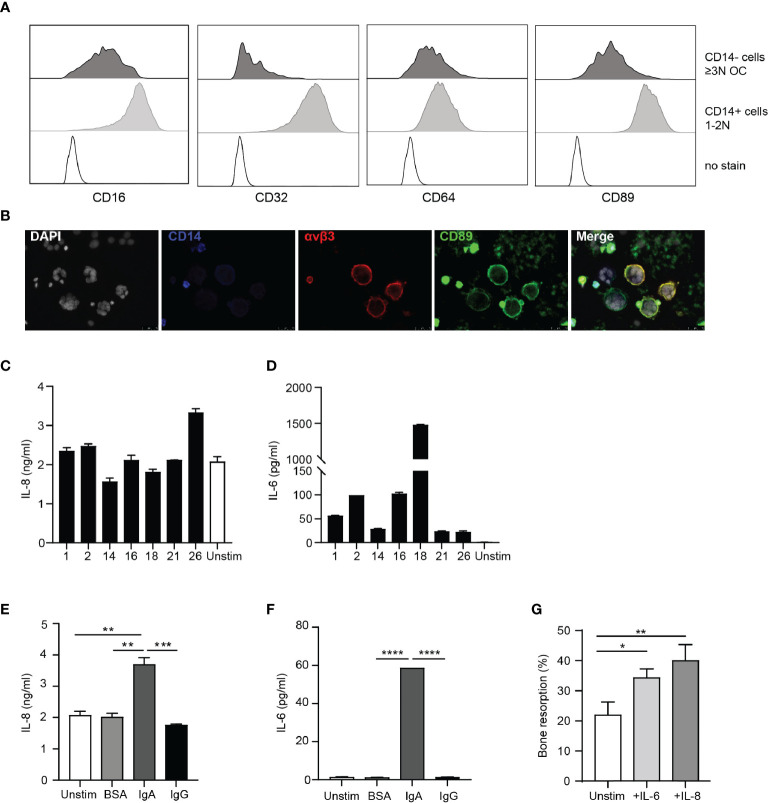
Osteoclasts are activated by IgA immune complexes. **(A)** Expression of CD16, CD32, CD64 and CD89 on CD14 negative OCs (upper panels; dark grey histograms) and remaining CD14^+^ cells (middle panels; light grey histograms) compared to no stain (lower panels; dotted black line histograms). **(B)** CD89 expression on CD14^-^αVβ3^+^ OCs. [White (DAPI) = nuclei, blue = CD14, red = αVβ3, green = CD89]. **(C, D)** IL-8 **(C)** and IL-6 **(D)** release by OCs stimulated with immune complexes isolated from synovial fluid of RA patients. **(E, F)** IL-8 **(E)** and IL-6 **(F)** release by unstimulated OCs (white bar) and after stimulation with BSA- (light grey bar), IgA- (dark grey bar) or IgG- (black bar) coated beads. **(G)** Quantification of bone resorption by OCs in presence of M-CSF and RANK-L (unstim, white bar) and added cytokines IL-6 (25 ng/ml, light grey bar) or IL-8 (10 ng/ml, dark grey bar). Data was analyzed using one way ANOVA with Tukey post-hoc; *p ≤ 0.05; **p ≤ 0.01; ***p ≤ 0.001; ****p ≤ 0.0001.

Next, we questioned whether presence of IgA autoantibodies also directly affects osteoclast activation. Human osteoclasts expressed intermediated levels CD16 (FcγRIII), CD64 (FcγRIII), CD89 (FcαRI), and low levels of CD32 (FcγRII) (**Figure 5A**, upper panels). Subsequently, CD89 expression on multinuclear CD14^-^αvβ3^+^ osteoclasts was confirmed with microscopy (**Figure 5B**). Remaining CD14^+^ cells expressed high levels of CD16, CD32 and CD89, and intermediate levels of CD64 (**Figure 5A**, lower panels).

To investigate the effect of IgA-FcαRI interactions on the bone resorptive capacity of osteoclasts, we separated CD14 negative osteoclasts from the remaining CD14 positive cells. Of the sorted osteoclasts >80% was alive and >95% was CD14 negative (**Supplementary Figure 3A**). The majority of sorted OC were multinucleated and express TRAcP (**Supplementary Figure 3B**). OCs activated with immune complexes isolated from synovial fluid of RA patients produced IL-8 (**Figure 5C**). However, similar levels of IL-8 were released by unstimulated osteoclasts. Production of IL-6 by osteoclasts after stimulation with SF immune complexes varied a lot between samples, but was not produced by unstimulated osteoclasts (**Figure 5D**). To investigate whether cytokine production was mediated by IgA or IgG, we stimulated OCs with IgA-, or IgG-coated beads. IgA-activated OCs produced significantly higher levels of IL-8 compared to unstimulated, BSA or IgG-activated OCs (**Figure 5E**). IL-6 was only produced by OCs after IgA activation (**Figure 5F**). Bone resorptive capacity of osteoclasts was significantly enhanced in the presence of IL-8 (p=0.0054) or IL-6 (p=0.03) (**Figure 5G**). These results support previous findings that IgA autoantibody levels in RA patients correlate with increased bone resorption. We now show that this can be a direct consequence of IgA-mediated activation of osteoclasts through the enhanced release of IL-8 and IL-6.

## Discussion

Bone remodeling in RA patients is disturbed, resulting in bone loss and eventually loss of function (10). In this study we demonstrated that IgA enhanced bone resorption through cytokines released by immune cells and by activating osteoclasts through FcαRI crosslinking. Our results show that IgA has a significant effect on bone resorption and in this way is likely responsible for worse pathology in RA patients.

The exact process of bone remodeling in RA patients is incompletely understood. It has been reported that multinucleated cells that express typical osteoclast lineage markers including TRAcP and cathepsin K are found at sites of bone erosion in RA patients (32). Multiple *in vivo* studies with osteoclast deficient mice support the critical role of osteoclasts in bone loss (33, 34). Bone loss in RA patients was strongly associated with presence of ACPA or anti-CarP antibodies (7, 35–37). In contrast to our results, it was shown that bone resorption was enhanced by ACPA IgG containing immune complexes *in vitro* (38). Increased numbers of bone resorbing cells were responsible for this enhanced bone resorption and was also observed in presence of non-ACPA IgG containing immune complexes (38). Presence of ACPA IgA or IgG and anti-CarP antibodies in sera were predictive for the development of RA (3, 7, 39), whereas only ACPA-IgA levels were associated with more active disease (40).

Next to osteoclasts, inflammatory cells present in the joint can indirectly contribute to bone resorption. Neutrophils are the most abundant immune cells present in the inflamed joint and can get highly activated by IgA and IgG resulting in the formation of NETs (18, 41). NET release was induced by immune complexes isolated from synovial fluid of RA patients. Variations in the abundancy of NET formation is likely due to different levels of autoantibodies in SF, influencing the level of neutrophil activation. NET release was not induced by immune complexes derived from serum, which may be due to less presence of immune complexes compared to synovial fluid. NETs can contribute to the autoimmune profile of RA patients, as myeloperoxidase can convert thiocyanate into cyanate, which is essential for carbamylation (42). Previously it was shown that NET formation was significantly increased in neutrophils stimulated with synovial fluid containing ACPAs compared to stimulation with ACPA-negative synovial fluids (43). Similarly we reported positive correlations between NET release and ACPA IgA and IgG levels. NETs may further contribute to the pathogenesis of RA as source of citrullinated autoantigens. In complex with ACPAs they can enhance the production of pro-inflammatory cytokines IL-6 and IL-8 by local (immune) cells (41). Recently a humanized anti-ACPA therapeutic antibody was described to inhibit NET formation by human neutrophils activated with SF of gout patients (44), supporting the importance of this autoantibody in the pathology of RA as well.

Deposition of immune complexes is a common pathogenic feature of several autoimmune diseases, including RA (45). Immune complexes containing antibodies against altered proteins are suggested to initiate a state of non-resolving inflammation in the synovial cavity, followed by RF containing immune complexes reactive to these initial immune complexes (46). However, in animal models it has been shown that immune complexes do not have to be specific against altered proteins to be able to initiate synovitis (47). In the synovium immune complexes can augment inflammation through complement activation (48), Fc receptor engagement (49), toll-like receptor binding (50), and stromal cell activation (51). Furthermore, ACPA containing immune complexes are suggested to be recognized by membrane epitopes (e.g. citrullinated vimentin) expressed by osteoclasts which promotes osteoclastogenesis (38). The glycosylation status of autoantibodies present in immune complexes, such as ACPA sialylation, additionally enhances the capacity to promote inflammation (52). We showed that incubation of monocytes stimulated with SF immune complexes resulted in the production of IL-6 and IL-8. The variable levels of cytokine release can be explained by the different levels of autoantibodies present in the immune complexes. IL-8 is an important chemokine for neutrophils (53) and positively correlated with the amount of infiltrated neutrophils in the inflamed synovium of RA patients (43). IL-8 levels produced by monocytes correlated with both IgA and IgG autoantibodies. These results are supported by previous work from our lab demonstrating that IL-8 is produced by monocytes after IgA or IgG activation (own unpublished data). Interestingly, osteoclasts produced significantly elevated levels of IL-6 and IL-8 after stimulation with SF complexes. The direct effect of IgA autoantibodies on osteoclast activity by blocking of FcαRI could not be formally determined, as FcαRI is continuously internalized and re-expressed. Blocking FcαRI during long term culture of osteoclasts is therefore not feasible. Nonetheless, only stimulation of osteoclasts with IgA-, but not IgG complexes resulted in enhanced production of IL-6 and IL-8, supporting that IgA autoantibodies in SF were responsible for the observed IL-6 and IL-8 production.

IL-8 after IgA, compared to IgG activation. Human CD14^+^ osteoclasts are described to express multiple Fc gamma receptors (54) and autoantibodies can bind osteoclasts specifically, thereby promoting osteolytic function *in vitro* (38). In this study we focused on CD14 negative osteoclasts, as the most mature bone resorbing cells (29), which have intermediate expression of FcγRI, FcγRIII, FcαRI, and low expression of FcγRII compared to CD14^+^ mononuclear cells.

IgG autoantibodies were described to enhance the production of osteoclastogenic factors, including IL-6, IL-8, TNF-α and IL-1β by monocytes and macrophages (13, 49). However, IgA-activation of monocytes induced higher levels of pro-inflammatory cytokines compared to IgG-activation (own unpublished data). Synovial fluid levels of IL-8 and IL-6 were significantly elevated in patients with high ACPA levels in their synovial fluid (43). ACPA-induced differentiation of osteoclasts was inhibited *in vitro* by a neutralizing IL-8 antibody. Furthermore, *in vivo* IL-8 inhibition reversed ACPA-induced bone loss in mice (55), supporting the importance of IL-8 in the pathology of RA in ACPA positive patients.

IL-6 production by monocytes only positively correlated with levels of ACPA IgA, but not ACPA IgG. These results are in line with previous work showing IL-6 release by monocytes upon IgA, but not IgG activation (own unpublished data). Similarly, osteoclasts only produced IL-6 after IgA activation. IL-6 significantly increased bone resorption by osteoclasts. *In vitro*, IL-6 was shown to enhance osteoclastogenesis through activation of RANKL on osteocytes and osteoblasts (56, 57). *In vivo*, IL-6 was necessary for inducing osteoclast activity and bone resorption (58, 59). Several clinical trials have shown the potential of the human anti-IL-6 blocking antibody tocilizumab in treating RA patients (60–62).

**Figure 6:**
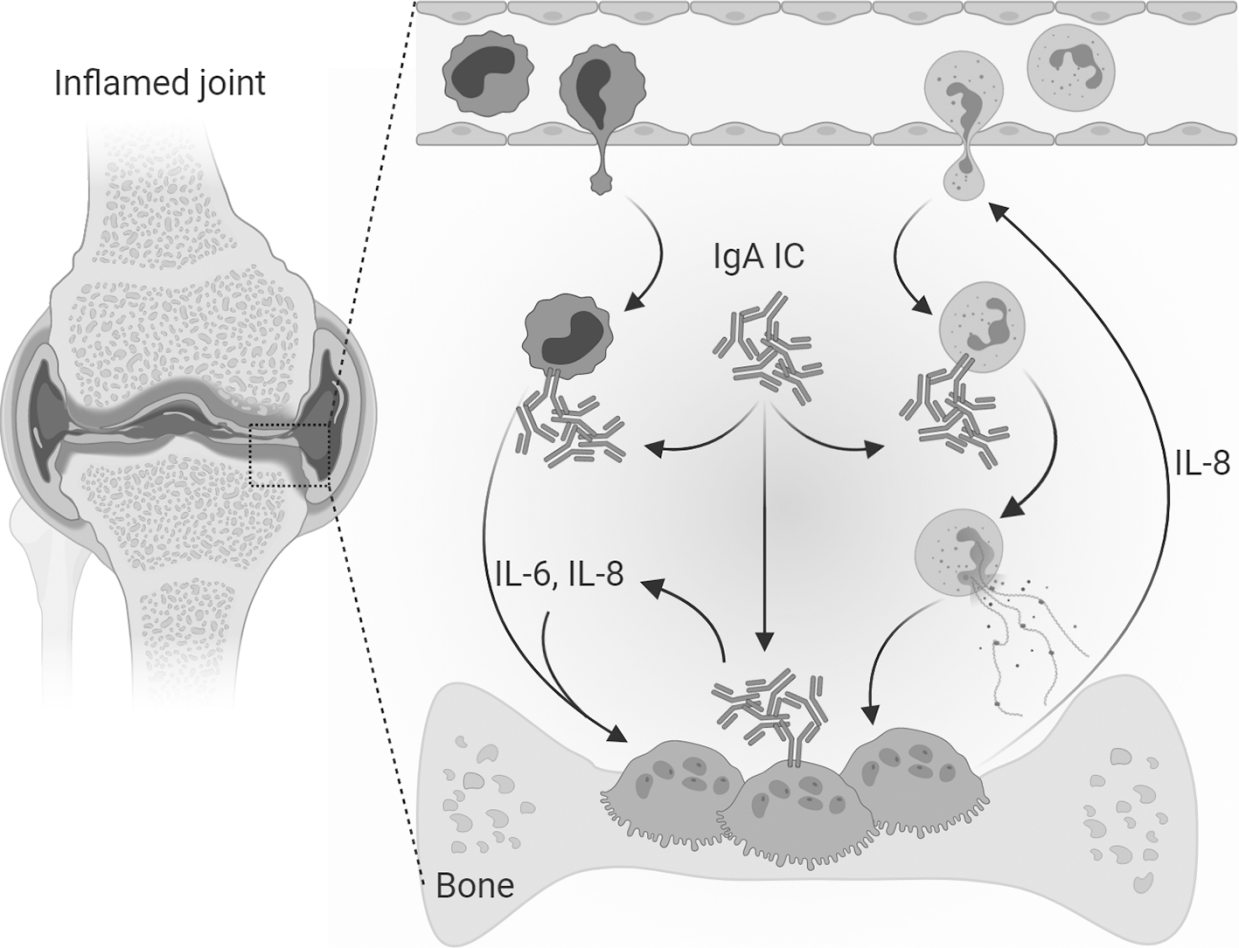
Mechanism of direct and indirect IgA-mediated osteoclast activation. In the inflamed joints of RA patients’ immune complexes consisting of IgA autoantibodies are present. Infiltrated neutrophils can form neutrophil extracellular traps (NETs) when activated with IgA-containing immune complexes. Monocytes activated with IgA-containing immune complexes produce high levels of IL-6 and IL-8 and enhance the bone resorptive capacity of osteoclasts. IgA-containing immune complexes can also directly target osteoclasts which express the Fc receptor for IgA (FcαRI). After IgA activation osteoclasts secrete IL-8 and IL-6, which are osteoclastogenic cytokines known to contribute to the formation of osteoclasts. Furthermore, IL-8 contributes to the chemoattraction and infiltration of neutrophils thereby enhancing the pro-inflammatory environment in the inflamed synovium of RA patients. Osteoclast secreted IL-8 could therefore both lead to auto stimulation and attraction of neutrophils to the inflamed area. [Created with BioRender.com].

In conclusion, we propose that IgA-FcαRI interactions on both infiltrated immune cells in the synovium and osteoclasts have an essential role in the pathogenesis of RA (**Figure 6**). IL-6 production after IgA activation of monocytes and osteoclasts can contribute to osteoclast formation, activity and their bone resorptive capacity. Elevated IL-8 production after IgA stimulation of monocytes and osteoclasts can contribute to the infiltration of neutrophils, thereby promoting an inflammatory environment. Furthermore it promotes osteoclast activity resulting in increased bone resorption. We anticipate that targeting IgA-FcαRI interactions is a promising therapeutic strategy to diminish bone resorption in RA patients.

## Data Availability Statement

The original contributions presented in the study are included in the article/**Supplementary Material**. Further inquiries can be directed to the corresponding author.

## Ethics Statement

The studies involving human participants were reviewed and approved by The Medial Ethical Committee of the Leiden University Medical Center (reference no. B15.003) and the Medical Ethical Committee of the Amsterdam University Medical Center (reference no. 2013.234). The patients/participants provided their written informed consent to participate in this study.

## Author Contributions

AB, MG, MD, and ME designed the experiments. AB, MG, and MD conducted the experiments. IJ contributed to the bone resorption assays. AB, MG, and MD analyzed the data. CL, TV, and ME provided scientific input. AB wrote the first draft of the manuscript. MG, MD, CL, TV, IJ, and ME revised the manuscript. ME supervised during the entire process. All authors contributed to the article and approved the submitted version.

## Funding

Netherlands Organization for Scientific Research (VICI 91814650).

## Conflict of Interest

The authors declare that the research was conducted in the absence of any commercial or financial relationships that could be construed as a potential conflict of interest.

## Supplementary Material

Supplementary Figure 1 Correlation between NET release after stimulation of neutrophils with immune complexes isolated from synovial fluid of RA patients with OD values of **(A)** ACPA IgA (left) or ACPA IgG (right), **(B)** anti-CarP IgA (left) or anti-CarP IgG (right) and **(C)** RF IgA measured in synovial fluid of RA patients.

Supplementary Figure 2
**(A)** Correlation between monocyte IL-6 and IL-8 release induced by immune complexes of RA SF. **(B, C)** Correlation between monocyte IL-6 release and **(B)** (left) anti-CarP IgA levels or (right) RF IgA, or **(C)** (left) ACPA IgG levels and (right) anti-CarP IgG levels. **(D)** Correlation between monocyte IL-8 release and (left) anti-CarP IgA levels and (right) anti-CarP IgG levels.

Supplementary Figure 3
**(A)** Gating strategy to check the purity of sorted osteoclasts. (left) Location of osteoclasts (OCs) on forward-side scatter area with adjusted laser settings to visualize large cells; (middle) viability of the OCs; (left) CD14 negative OCs. **(B)** Multinucleated osteoclast TRAcP expression (purple) 24 hours after sorting [Blue (DAPI) = nuclei].
