# Supplementary figures and images for: IgA Immune Complexes Induce Osteoclast-Mediated Bone Resorption
Source: Front Immunol. 2021 Jul 1;12:651049. doi: 10.3389/fimmu.2021.651049 (PMC8281931; doi:10.3389/fimmu.2021.651049)

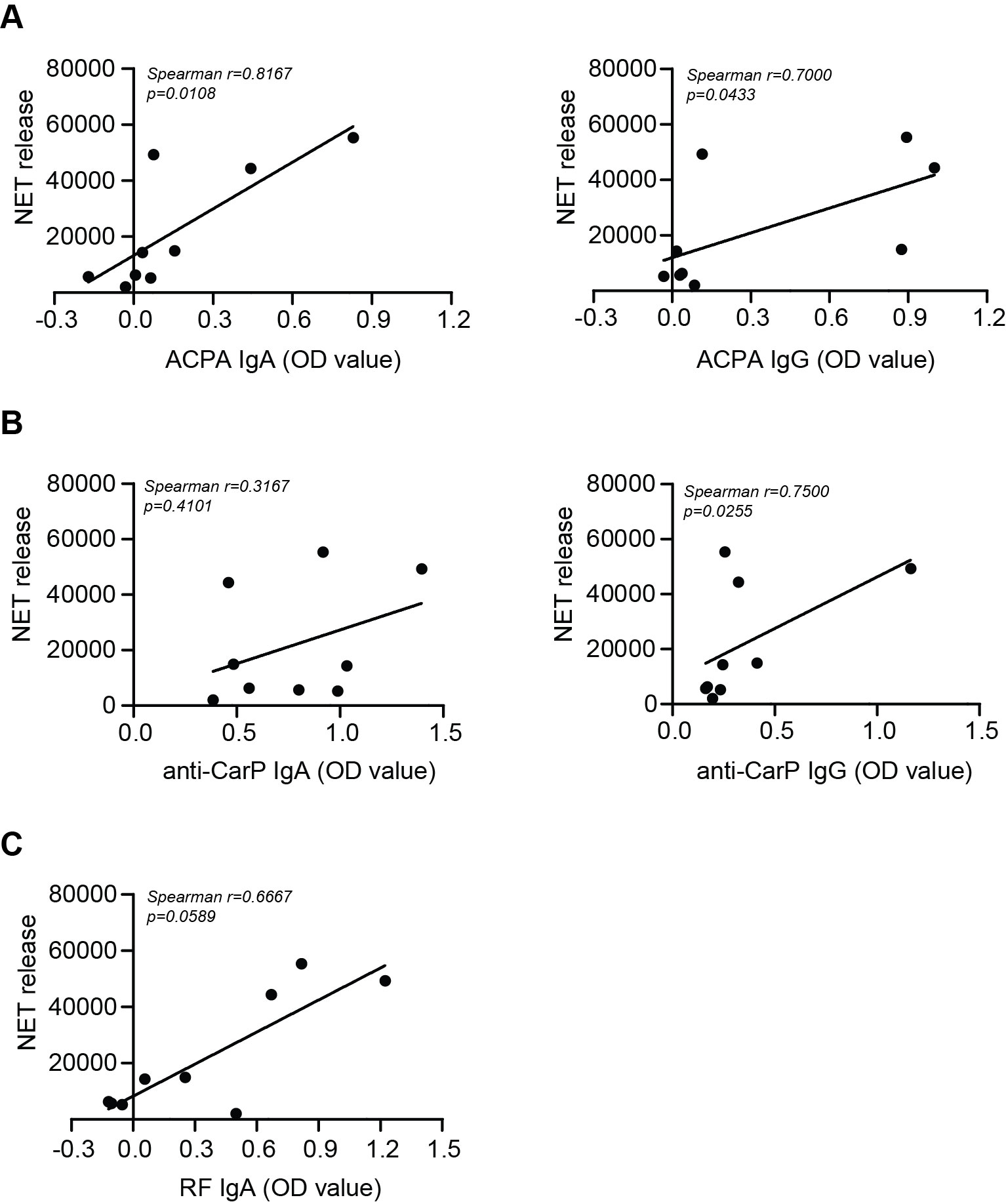

Supplement: Supplementary Figure 1 — Correlation between NET release after stimulation of neutrophils with immune complexes isolated from synovial fluid of RA patients with OD values of (A) ACPA IgA (left) or ACPA IgG (right), (B) anti-CarP IgA (left) or anti-CarP IgG (right) and (C) RF IgA measured in synovial fluid of RA patients. [file Image_1.jpg]

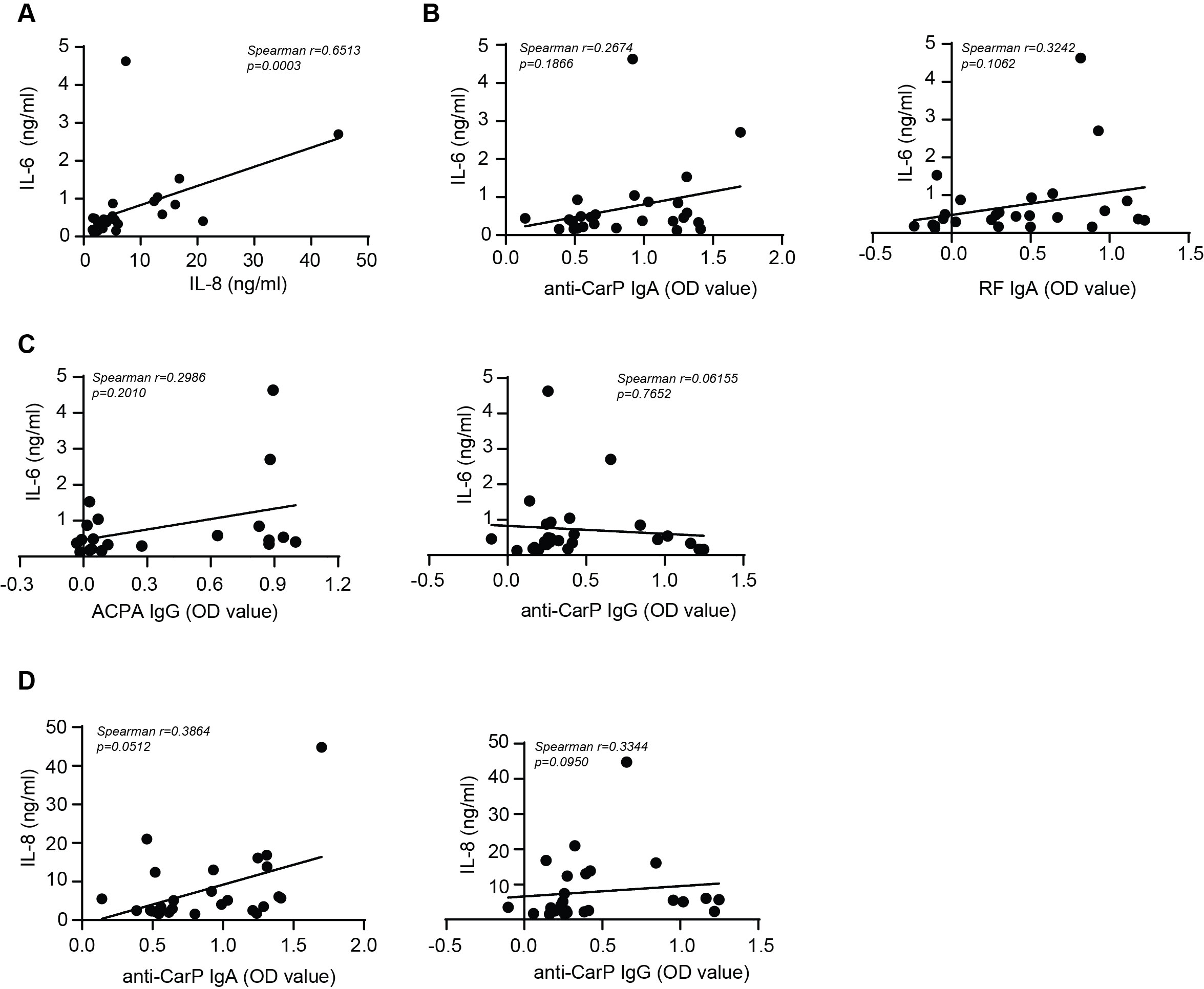

Supplement: Supplementary Figure 2 — (A) Correlation between monocyte IL-6 and IL-8 release induced by immune complexes of RA SF. (B, C) Correlation between monocyte IL-6 release and (B) (left) anti-CarP IgA levels or (right) RF IgA, or (C) (left) ACPA IgG levels and (right) anti-CarP IgG levels. (D) Correlation between monocyte IL-8 release and (left) anti-CarP IgA levels and (right) anti-CarP IgG levels. [file Image_2.jpg]

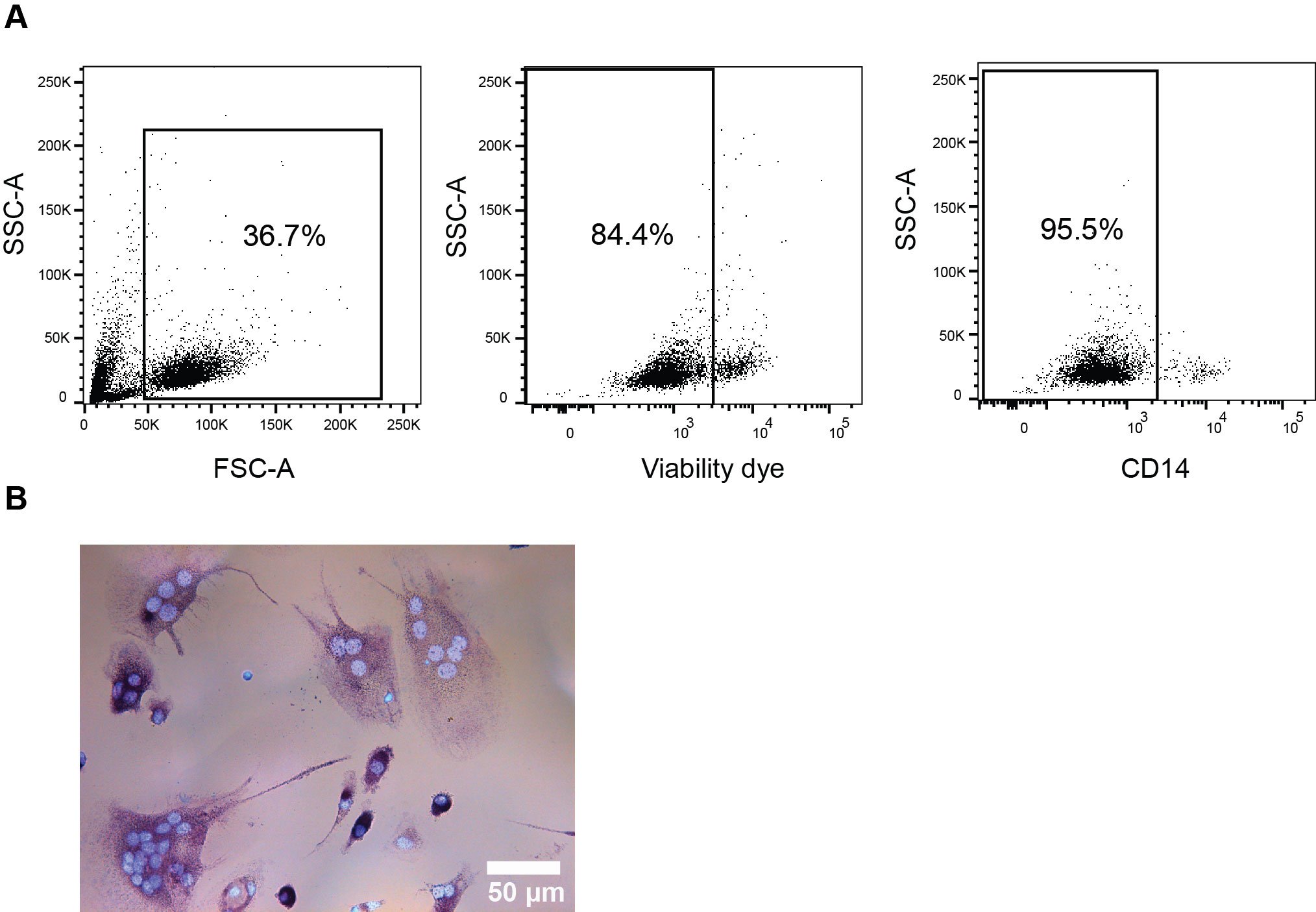

Supplement: Supplementary Figure 3 — (A) Gating strategy to check the purity of sorted osteoclasts. (left) Location of osteoclasts (OCs) on forward-side scatter area with adjusted laser settings to visualize large cells; (middle) viability of the OCs; (left) CD14 negative OCs. (B) Multinucleated osteoclast TRAcP expression (purple) 24 hours after sorting [Blue (DAPI) = nuclei]. [file Image_3.jpg]
